# Enriched Aptamer Libraries in Fluorescence-Based Assays for *Rikenella microfusus*-Specific Gut Microbiome Analyses

**DOI:** 10.3390/microorganisms11092266

**Published:** 2023-09-09

**Authors:** Yiting Zhang, Hu Xing, Grigory Bolotnikov, Markus Krämer, Nina Gotzmann, Uwe Knippschild, Ann-Kathrin Kissmann, Frank Rosenau

**Affiliations:** 1 Institute of Pharmaceutical Biotechnology, Ulm University, Albert-Einstein-Allee 11, 89081 Ulm, Germany; yiting.zhang@uni-ulm.de (Y.Z.); hu.xing@uni-ulm.de (H.X.); grigory.bolotnikov@uni-ulm.de (G.B.); markus-1.kraemer@uni-ulm.de (M.K.); nina.gotzmann@uni-ulm.de (N.G.); ann-kathrin.kissmann@uni-ulm.de (A.-K.K.); 2 Department of General and Visceral Surgery, Surgery Center, Ulm University, Albert-Einstein-Allee 23, 89081 Ulm, Germany; uwe.knippschild@uniklinik-ulm.de; 3 Max-Planck-Institute for Polymer Research Mainz, Ackermannweg 10, 55128 Mainz, Germany

**Keywords:** DNA aptamer, biosensor, Cell-SELEX, in vitro diagnostic, *Rikenella microfusus*

## Abstract

*Rikenella microfusus* is an essential intestinal probiotic with great potential. The latest research shows that imbalance in the intestinal flora are related to the occurrence of various diseases, such as intestinal diseases, immune diseases, and metabolic diseases. *Rikenella* may be a target or biomarker for some diseases, providing a new possibility for preventing and treating these diseases by monitoring and optimizing the abundance of *Rikenella* in the intestine. However, the current monitoring methods have disadvantages, such as long detection times, complicated operations, and high costs, which seriously limit the possibility of clinical application of microbiome-based treatment options. Therefore, the intention of this study was to evolve an enriched aptamer library to be used for specific labeling of *R. microfusus*, allowing rapid and low-cost detection methods and, ultimately the construction of aptamer-based biosensors. In this study, we used *Rikenella* as the target bacterium for an in vitro whole Cell-SELEX (Systematic Evolution of Ligands by EXponential Enrichment) to evolve and enrich specific DNA oligonucleotide aptamers. Five other prominent anaerobic gut bacteria were included in this process for counterselection and served as control cells. The aptamer library R.m-R13 was evolved with high specificity and strong affinity (K_d_ = 9.597 nM after 13 rounds of selection). With this enriched aptamer library, *R. microfusus* could efficiently be discriminated from the control bacteria in complex mixtures using different analysis techniques, including fluorescence microscopy or fluorometric suspension assays, and even in human stool samples. These preliminary results open new avenues toward the development of aptamer-based microbiome bio-sensing applications for fast and reliable monitoring of *R. microfusus*.

## 1. Introduction

Billions of microbial cells are on and in the human body and form the human microbiome, which is inextricably linked to health and disease and plays a vital role in both. Research on the intestinal flora has developed in recent years, revealing definitive evidence of its association with digestive tract disorders and neurological, endocrine, cardiovascular, reproductive, and other diseases [1,2,3,4]. *R. microfusus* is an important gut bacterium, and its abundance has been found to have a close relationship with the onset of numerous disorders, which has recently become a hot research topic. It was first isolated by Kaneuchi and Mitsuoka in 1978 from many fecal or cecum specimens from calf, chicken, and Japanese quail [5]. The impact of *Rikenella* on human health appears to be multifaceted and can be both positive and negative, depending on the context of individual diseases and disorders of the patient. A still increasing number of studies currently deliver a diffuse picture on the role of these generally low-abundant bacteria but imply distinct effects on prominent disorders, although some data are based on studies in animals only. The abundance of *Rikenella*, for example, in the intestine of human patients with inflammatory bowel disease (IBD), has been found to be significantly suppressed compared to healthy patients [6,7,8]. In mice with autoimmune disease, levels of *Rikenella* were also substantially lower than expected [9]. *Rikenella* appears to be a crucial gut microbiome member and potential probiotic that is probably essential for reducing intestinal inflammation [10,11]. Short-chain carboxylic acids, including succinic acid, propionic acid, acetic acid, and alcohols, as typical metabolites of *Rikenella*, can effectively improve the intestinal flora environment and enhance microbial abundance [6]. Evidence suggests that *Rikenella* can help intestinal cells form tight junctions, which can help epithelial cells differentiate, thereby strengthening the intestinal barrier [12,13]. As the regulation of intestinal microflora is an essential factor in the fight against diarrhea, another study further demonstrated that *Rikenella* is also one of the most effective anti-diarrheal probiotics, with its abundance negatively correlated with the diarrhea index [14]. In mouse allergy models, the allergic symptoms were significantly improved by reducing the abundance of *Rikenella* [15]. In another group of mouse obesity models, the abundance of *Rikenella* in the intestine of these mice was significantly lower than that of lean mice [16]. Interestingly, increasing the amount of *Rikenella* in the intestine of mice markedly decreased insulin resistance in type 2 diabetes [17,18,19]. Intestinal barrier dysfunction and bacterial translocation can lead to inflammation in chronic kidney disease (CKD) [20,21]. Early renal complications caused by diabetes can be prevented by modulating the intestinal flora. In one study, intestinal permeability and inflammation in mice were improved by altering the intestinal microbiota, including *Rikenella*, through pharmacological treatment [22]. In addition, *Rikenella* is significantly dysregulated in depressed patients, and receiver operating characteristic (ROC) curves show that *Rikenella* may be a potential biomarker for depression-like behavior in mice [23,24]. What is more intriguing is that, while certain microflorae are diminished or missing in many diseases, they are unusually plentiful in healthy individuals, suggesting that they may serve as a target or biomarker for the therapy of various disorders. Certain retinal neurodegenerative diseases are also associated with gut microbial homeostasis. *Rikenella* is widespread in healthy gut microbiomes but is essentially absent in mouse models of retinal disease [25,26]. *Rikenella* is abundant in HIV-negative subjects but progressively declines or depletes in untreated HIV patients [27,28,29,30]. Metabolites produced by specific microbiota are also involved in several lipid metabolisms, membrane transport, and other pathways that are considered relevant to the pathogenesis of Alzheimer’s disease (AD) [31]. *Rikenella* was scarce in AD mice, but this was quickly reversed after gut flora-targeted therapy, suggesting that *Rikenella* may be a potential target for the treatment of AD [32]. Gut flora can be involved in the treatment of AD-related cognitive impairment by controlling the dysregulation of pyrimidine metabolism, and *Rikenella* may therefore be a significant biological marker for AD [33].

Using the Cell-SELEX method, aptamer libraries can be obtained with high affinity and selectivity after repeated selection in vitro. Aptamers offer numerous benefits over antibodies, including low cost, low molecular weight, non-immunogenicity, and simplicity of modification. As a result, they have attracted much attention in recent years for use in drug administration, targeted therapeutic, and diagnostic applications [34]. Since its initial application in 1990, SELEX technology has evolved. Significant advances have been achieved in the design, construction, and use of aptamers, such as making the screening process more efficient, less costly, and less time-consuming [35,36,37]. SELEX is an iterative process in which the screening procedure includes the binding of the aptamers, the isolation of target-bound molecules, and subsequent PCR amplification. This results in the culmination of sequences with target affinity and efficient enrichment of a library of nucleic acid aptamers with increasing affinity and specificity toward the target structure (i.e., cells in whole Cell-SELEX) after repeated rounds of evolution with increasing selection pressure by harshening the binding/washing conditions [38,39].

**Figure 1:**
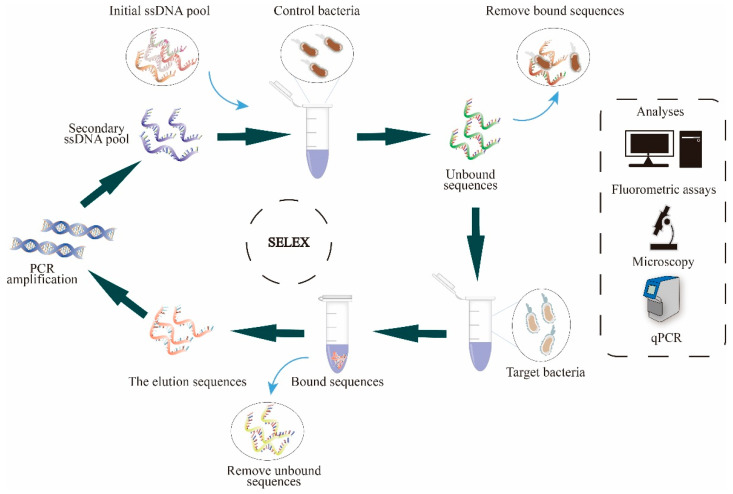
SELEX is a directed evolution process performed completely in the laboratory leading to oligonucleotide DNA or RNA molecules (aptamers) with binding affinity to target structures. In principle, a (commercial) starting library of up to 10^14^ individual sequences is used for binding to the desired target. In an iterative process of binding, washing off non-binders, harvesting of binders, and their amplification by PCR, the affinity and specificity of the aptamers will increase (or “evolve”). A prerequisite is a certain selection pressure toward better binding, which can gain stringency by reducing the amount of aptamers provided, increasing the harshness of washing, and using so-called counter-selection steps using non-specific targets like other gut bacteria and/or non-aptamer nucleic acids as well as blocking proteins like casein. Here, the selection pressure and thus the efficiency of evolution were enhanced by incubating the initial library with a mixture of control bacteria, including *A. muciniphila*, *A. stercoricanis*, *B. producta*, *R. intestinalis,* and *P. distasonis,* during the initial rounds of selection. Counter-SELEX was used to eliminate aptamers unbound to target bacteria. Subsequently, the aptamers obtained in the previous evolution round were incubated with the target bacterium, *R. microfusus*, for target SELEX. After repeating several rounds of screening/selection, we obtained aptamer eluates to obtain new pools of enriched DNA by PCR amplification. Ultimately, we analyzed the enriched aptamer libraries we obtained from those specific binding target cells in various ways, including fluorescence analysis, fluorescence microscopy observation, and qPCR quantitative analysis.

In our previous studies, we have facilitated our real-time monitoring of the screening/selection process by Cy5 fluorescent-labeled DNA aptamers and direct measurement of aptamer binding to target cells by changes in fluorescence intensities on the cells. This technique we introduced as the FluCell-SELEX [40,41,42,43,44]. In order to boost the SELEX screening pressure and improve stringency, a mixture of five other intestinal bacteria was used in a counter-selection, including *A. muciniphila*, *A. stercoricanis*, *B. producta*, *R. intestinalis,* and *P. distasonis* in this study (Figure 1). The final aptamer library, R.m-R13, has a strong affinity for *R*. *microfusus* and enables efficient quantification of this bacterium in the mixed model intestinal flora. As far as we know, we are the first to isolate and directly use an aptamer library to identify or label this probiotic strain, providing a solid foundation for the subsequent development and optimization of more accessible and cost-effective assays and biosensors for the clinical detection and subsequent fast and precise quantification of *Rikenella*.

## 2. Results

**Figure 2:**
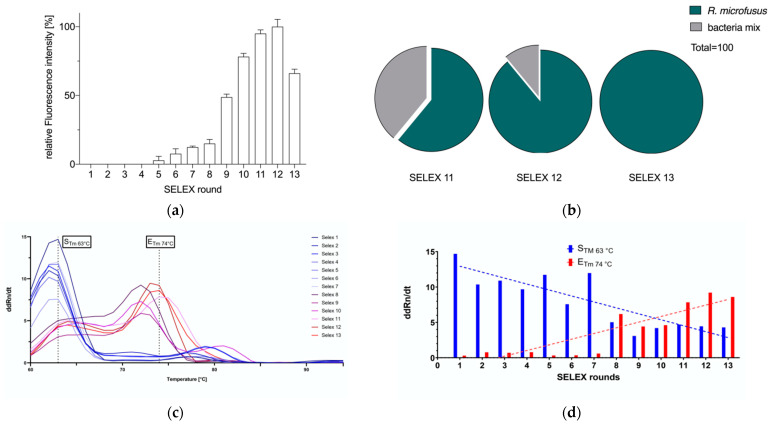
(**a**) Affinity and (**b**) specificity assay for *R. microfusus*, using the aptamer library R.m-R13. The specificity of the enriched aptamer library obtained in rounds 11–13 increased progressively compared to the mixture of the other five intestinal bacteria *B. producta*, *A. muciniphila*, *R. intestinalis*, *A. stercoricanis,* and *P. distasonis*. All experiments were performed using five pmol aptamer and 10^8^ cells, and fluorescence intensity was measured at 635 nm excitation and 670 nm emission. All experiments were performed in three sessions (*N* = 3). (**c**) Melting curves of the prominent temperature peaks, including S_Tm_ at the start and E_Tm_ at the end of SELEX; (**d**) peak shift analyses for S_Tm_ and E_Tm_, and linear regression fitted to ddRn/dT (fluorescence change/temperature change) with different SELEX rounds.

In 13 rounds of SELEX, we have evolved the particular DNA aptamer library R.m-R13 against the gut bacterium *R. microfusus* using the FluCell-SELEX technique. We performed positive selection for *R. microfusus* in the first four rounds to enhance the number of specific DNA aptamers by compensating for the dearth of single specific aptamers, which was a considerable limitation of the procedure in the initial library. From the fifth round onwards, adding a counter-selection step to the procedure using the negative control bacterial mixture prior to the positive selection resulted in a more efficient development of the specific aptamer library, as indicated by the onset of a positive fluorescence signal starting with the fifth SELEX cycle (Figure 2a). Since rounds 5–8 proceeded steadily but rather slowly, the number of counter-selection bacteria increased the pressure from round 9 onwards. As a result, the binding intensity of the aptamer library increased drastically. In rounds 11 and 12, the fluorescence signal leveled off but was reduced by 25% after the 13th round, and hence we considered the screening process completed (Figure 2a) [45]. Using the ensemble of the five negative control gut bacteria, we then determined the specificity of the enriched aptamer libraries for *R. microfusus* surface targets in rounds 11–13, respectively.

The progressive increase in library specificity indicated that the ability of the aptamer library to distinguish between *R. microfusus* and the control strains was effectively improved. The aptamer library R.m-R13 obtained in the final productive round (13) showed the highest level of specificity, although the affinity was somewhat weaker than in round 12 (Figure 2b). Further, the melting curves of the aptamer libraries originating from the SELEX rounds were analyzed in qPCR reactions. Tm indicates the average melting temperature profile, and the melting temperatures S_Tm_ and E_Tm_ at the start and end of the SELEX were determined, respectively. During the SELEX process, the library melting temperature rose from 63 °C (S_Tm_) to 74 °C (E_Tm_) (Figure 2c). The effective increase in maximum library melting temperature originates from the enrichment of DNA aptamers with high GC content in the library, which generally tend to have better secondary structure stability and can provide more potential hydrogen bonds toward target atoms. In order to compare the degree of shift in the melting curve during the screening process, we quantified the relative ddRn/dT values for each screening round at the peak temperatures of S_Tm_ and E_Tm_ to provide a measure of the Tm shift in the screening process, where the ddRn/dT values gradually decreased at S_Tm_ and, in contrast, increased and eventually stabilized at E_Tm_ (Figure 2d).

**Figure 3:**
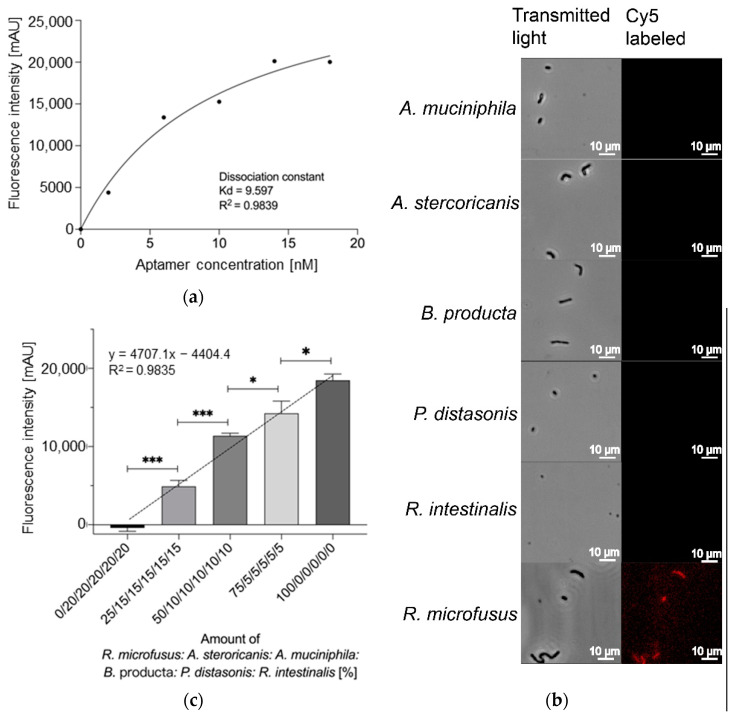
(**a**) Determination of the dissociation constant for the aptamer library R.m-R13 with a K_d_ of 9.597 nM and a deviation of 0.9839 in the coefficient of determination R^2^. (**b**) Fluorescence microscopy observations of the aptamer library R.m-R13 bound to *R. microfusus*, *B. producta*, *A. muciniphila*, *R. intestinalis*, *A. stercoricanis,* and *P. distasonis*. Cy5-labeled R.m-R13 displayed a strong fluorescent signal bound to *R. microfusus*, whereas the other five bacteria as controls delivered, as expected, no signals. (**c**) Quantification of the aptamer library R.m-R13 on *R. microfusus* in a mixture that included *B. producta*, *A. muciniphila*, *R. intestinalis*, *A. stercoricanis,* and *P. distasonis*, mixed in different proportions at the same OD. All experiments were performed using five pmol aptamers in 10^8^ cells, and all experiments were performed in three runs (*N* = 3). *p* values < 0.05 were considered significant. * denotes *p* < 0.05, *** denotes *p* < 0.001.

To quantitatively evaluate the strength of binding affinity of the aptamer library R.m-R13, we further obtained the dissociation constant (K_d_) of R.m-R13 by co-incubating quantitative *R. microfusus* with different concentrations of the Cy5-labeled aptamer library (Figure 3a), which was determined to have a K_d_ value of 9.597 nM, indicating that the selected aptamer R.m-R13 has a high affinity for *R. microfusus*. At the same optical density settings, fluorescence microscopy was used to identify the binding of the aptamer library R.m-R13 to the target bacterium *R. microfusus* and five other enteric bacteria, including *B. producta*, *A. muciniphila*, *R. intestinalis*, *A. stercoricanis,* and *P. distasonis*. The results showed that the aptamer library R.m-R13 exhibited intense labeling of *R. microfusus* while failing to label the other five control bacteria (Figure 3b). Next, we analyzed the ability of the aptamer library to retrace *R. microfusus* in different proportions of mixed bacteria, and the fluorescence intensity revealed a significant positive linear correlation with the number of target bacteria (R^2^ = 0.9835), and the aptamer library was able to track an increase in *R. microfusus* numbers successfully (Figure 3c).

**Figure 4:**
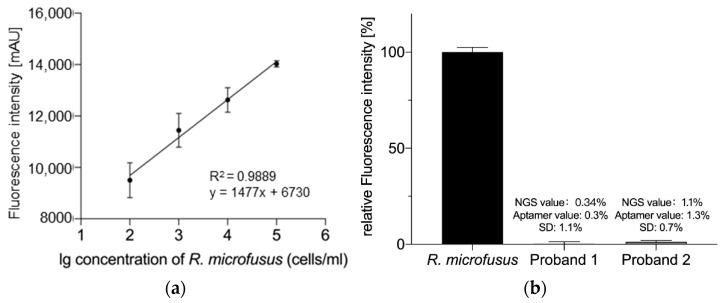
(**a**) Sensitivity assay of the aptamer library R.m-R13. In a 1 mL reaction system containing five pmol of the aptamer library, R.m-R13 showed a significant positive correlation between the number of bacteria and fluorescence intensity for *R. microfusus* numbers greater than 10^2^ (R^2^ = 0.9889), and therefore, the minimum detection limit for the aptamer library was determined to be 10^2^ cells/mL. (**b**) Comparison of *Rikenella* abundance measurements in fecal samples from the aptamer library R.m-R13 and 16s rRNA NGS (see Tables S2 and S3, Supplementary Material). “NGS values” represent the actual *Rikenella* content in fecal bacteria. The “Aptamer value” represents the amount of *Rikenella* in fecal bacteria determined using the aptamer library R.m-R13. Error bars indicate the standard deviations of the experiments conducted in triplicate.

To further characterize the aptamer library R.m-R13 as binding entities for biosensing applications, we evaluated the detection limit of the library. Aptamers were quantified by co-incubation with various *R. microfusus* numbers to generate a standard fluorescence intensity curve as a function of the bacterial count (Figure 4a).

With *R. microfusus* levels of more than 10^2^ in 1 mL samples, the fluorescence intensity was positively correlated with the number of bacteria. (R^2^ = 0.9889). Considering the fact that there are plenty of bacteria in fecal samples, it is reasonable to assume that the aptamer library R.m-R13 could detect the abundance of *R. microfusus* from even a low amount of fecal samples. To test this hypothesis, we selected feces from two healthy volunteers (Proband 1 and Proband 2 in Figure 4b). We used the aptamer library R.m-R13 and NGS to determine the abundance of *Rikenella* in the subject samples, respectively. First, we set the relative fluorescence intensity of the control *R. microfusus* to 100% by the assay using the aptamer library.

In contrast, the relative fluorescence intensity of the experimental group was expressed as a percentage of *Rikenella* in the Proband fecal samples, with fluorometric results of 0.3% and 1.3%, respectively. The corresponding standard deviations were individually 1.1% and 0.7%. Additionally, by NGS, we obtained 0.34% and 1.1% of *Rikenella* in fecal bacteria for Proband 1 and Proband 2, respectively (see Tables S2 and S3, Supplementary Material). As a result, we believe that the aptamer R.m-R13 can fully satisfy requirements for both the sensitivity and accuracy of *Rikenella* detection in practical applications, which also provides a robust theoretical foundation for installing the biochip in the future. 

The correct abundance of *R. microfusus*, one of the most critical potential intestinal bacteria, has been associated with intestinal diseases, metabolic diseases, HIV, and AD, and therefore, quantitative monitoring of the composition of intestinal flora may be crucial for the prevention and potential therapy of these diseases [11,21,30,31]. However, existing intestinal flora assays, such as 16S rRNA gene sequencing and bacterial group/species quantitative polymerase chain reaction (qPCR), have the considerable disadvantages of being time-consuming, complex, and costly, and they often require specialized laboratories and personnel (for a comparison of techniques, see Table 1).

Developing a rapid and easy-to-use method to monitor intestinal flora is a challenging but attractive investment in the future of medical microbiome research. Therefore, it is urgent to develop a quick and convenient way to monitor intestinal flora and its changes [46], where the use of biosensors enables rapid, simple, low-cost, and quantitative detection. Aptamers as nucleic acid ligands can be repeatedly denatured and denaturalized under heat, different salt concentrations, and metal chelators. They can be modified and solidified, are highly stable, and are easily labeled with the target moiety, thus gradually replacing antibodies as binding entities in bioassays in recent years [47,48].

Living cells, exhibiting a natural 3D structure of epitopes on their cell surfaces, are targets in the whole Cell-SELEX variant used here. Compared to inactivated cells as targets, this natural conformation of surface molecules is crucial for productive aptamer screening [49]. Whole-cell screens tend to have higher fidelity than screens using purified proteins and are therefore more versatile [50]. The aptamer library R.m-R13, obtained after 13 rounds of Cell-SELEX, allows the specific detection of *Rikenella* in complex intestinal flora mixtures and human feces. R.m-R13 exhibits a low dissociation constant (K_d_ = 9.597 nM), ensuring a high affinity.

Furthermore, in fluorescence assays, the aptamer library R.m-R13 clearly showed a binding preference for the target bacterium *R. microfusus* compared to controls *B. producta*, *A. muciniphila*, *R. intestinalis*, *A. stercoricanis*, and *P. distasonis* demonstrating its *R. microfusus*-specific recognition potential and possible use as a diagnostic tool. Compared to the levels of *Rikenella* in human fecal samples identified by 16S rRNA next-generation sequencing (0.34% and 1.1% for Proband 1 and Proband 2, respectively), the levels of *R. microfusus* obtained by fluorescence analysis of the aptamer R.m-R13 were broadly consistent with the NGS assay results (0.3% and 1.3%). However, NGS needs a method for obtaining valid, complete, and reliable bacteria numbers categorized below the genus [44,51]. Therefore, we could not determine the actual *R. microfusus* content from the NGS assay results alone. As we did not evaluate the specificity of the aptamer library R.m-R13 for another subgenus of *Rikenella*, we suggest that aptamer binding occurs at the level below the genus, and we, therefore, consider that the results of the aptamer library assay correspond to the levels of all bacteria of the genus *Rikenella* in fecal samples.

We currently aim to develop electronic biosensors for quantifying and differentiating gut bacteria as biomarkers involving enriched aptamer libraries as binding entities on graphene FET sensors. We have previously developed such electronic aptasensors for the diagnostic differentiation of holo from apo forms of the human retinol binding protein 4 (RBP4), which allowed sensitive and highly reproducible quantitative measurements of the target protein, principally demonstrating the feasibility of aptamer-mediated gFET-based diagnostics of even delicate structural differences in biomolecules [52]. Based on our experience with SELEX processes against microbial cells, including different pathogens and (probiotic) gut bacteria, and the subsequent characterization of the enriched libraries (and individual aptamers selected from these libraries), we are confident that this sensor concept will also be fully reproducible with bacteria as analytes. Nevertheless, this has to be carefully examined in detail with respect to the intention to enable multiplex analyses, allowing parallel quantification of free, definable sets of gut bacteria within individual stool samples without significant cross-reactivities. In-depth characterization of the performance of the enriched aptamer library presented here (and the other already available libraries) will also allow for the estimation of the potential of individual aptamers isolated from the libraries by bioinformatic techniques to optimize sensitivity and specificity for reliable (diagnostic) measurements. *Rikenella* can serve as a potential target and biomarker for a set of diseases, and constant monitoring of its abundance may help to develop not only a better understanding of dysbiosis in health and disease development but may also facilitate dedicated probiotic treatments of bacterial imbalances as reasons for disease. The successful development of the aptamer library R.m-R13, which explicitly labels *Rikenella*, represents the first and most important milestone on our way to a specific *R. microfusus* aptasensor, thereby paving new avenues for new microbiome monitoring techniques as a promising amendment to the portfolio of diagnostic tools in the near future.

## 3. Materials and Methods

### 3.1. Cells and Culture Conditions

The bacteria strains *R. intestinalis* (DSM-14610), *P. distasonis* (DSM-29491), *A. muciniphila* mucT (DSM-22959), *A. stercoricanis* (DSM-13633), *B. producta* (DSM-29491), and *R. microfusus* (DSM-15922) were cultivated in Schaedler-Bouillon Medium (Carl Roth GmbH + Co. KG, Karlsruhe, Germany) at 37 °C under anaerobic conditions.

### 3.2. Materials

A random sequence library (TriLink BioTechnologies, Inc, San Diego, CA, USA) was synthesized and purified. The sequence was as follows: 5′-TAGGGAAGAGAGAGAGGACATATGAT-N_(40)_-TTTGACTAGTACATGACCACTTGA-3′ (Table 2). The initial library consists of three parts: a random sequence of 40 nucleotides in the middle and a fixed sequence of 23 nucleotides at each end that can be precisely and complementarily paired with primers, including Cyanine 5-labeled forward primers, 5′-Cy5-TAGGGAAGAAGGAGAGAGATGATA-3′, and phosphate-labeled reverse primers 5′-phosphate-TCAGTGTCATGTACTAGTCAA-3′ (Biomers.net GmbH, Ulm, Germany) (Table 2).

### 3.3. Cell-SELEX

Cell SELEX includes counter SELEX as well as target SELEX. Counter SELEX uses bacteria mix, including *B. producta*, *A. muciniphila*, *R. intestinalis*, *A. stercoricanis,* and *P. distasonis*, to obtain aptamers that do not bind to bacteria combination, which can be further incubated with *R. microfusus* by target SELEX to acquire a library of aptamers that specifically bind to *R. microfusus*. Cell SELEX can be divided into the following steps.

#### 3.3.1. Cell Pretreatment

Six bacteria were incubated under anaerobic conditions for 10–15 h, centrifuged at 9000× *g* for 1 min, and the OD_600_ of the bacterial solution was adjusted to 1 after washing once with 1 × DPBS buffer.

#### 3.3.2. Aptamer Library Activation

A total of 0.5 nmol of the initial library or 10/5 pmol of the ssDNA library was added to 500 µL of 1 × DPBS and incubated at 95 °C for 5 min, followed by an ice bath for 5 min. The final incubation was at room temperature for 20 min to ensure that the aptamers had the same 3D structure.

#### 3.3.3. Selection

The activated library was incubated with the control five-cell mixture for 1 h at 37 °C and centrifuged at 3000× *g* for 2 min. The supernatant was pipetted into a new 1.5 mL centrifuge tube, BSA (100 mg/mL) and tRNA (10 mg/mL) were added, gradually increasing as the screen progressed to improve stringency, and subsequently incubated with *R. microfusus* for 30 min at 37 °C. The supernatant was removed by centrifugation at 3000× *g* for 2 min and the pellet was washed with 1 × DPBS to discard unbound nucleic acids (see Table S1, Supplementary Material).

#### 3.3.4. Elution

The washed bacteria pellet was resuspended in 100 µL of 1 × DPBS and incubated for 5 min at 95 °C to disrupt the aptamer 3D structure and separate it from the cells. The supernatant was then collected by centrifugation at 11,000× *g* for 1 min.

#### 3.3.5. Acquisition of Secondary Libraries

SELEX collected aptamers by PCR amplification in a total reaction volume of 50 µL: 10 µL 5× Herculase II reaction buffer, 1.25 µL dNTPs 10 mM, 0.125 µL Cy5-Primer (biomers.net GmbH, Ulm, Germany) 10 µM, 0.125 µL phosphate-Primer (biomers.net GmbH, Ulm, Germany) 10 µM, 0.25 µL Herculase II Fusion DNA Polymerase (Agilent Technologies, Inc., Santa Clara, CA, USA), 1 µL template DNA, and water.

The amplification conditions were as follows: 2 min at 95 °C, followed by 25 cycles of 95 °C for 30 sec, 56 °C for 30 sec, 72 °C for 10 sec, and a final extension of 72 °C for 2 min. The obtained PCR products were purified using an optimized purification kit (MACHEREY-NAGEL GmbH & Co. KG, Düren, Germany). The obtained PCR products were purified by using an optimized purification kit (MACHEREY-NAGEL GmbH & Co. KG, Düren, Germany), catalyzed by λ-exonuclease to single-stranded DNA (New England Biolabs, Ipswich, MA, USA), and the treated samples were purified by an optimized PCR purification kit (MACHEREY-NAGEL GmbH & Co. KG, Düren, Germany) to obtain new DNA pools for subsequent SELEX. To get greater yields from the PCR products and subsequent ssDNA purification, 1.5 times more isopropanol and 10 µL of NaAc solution (pH = 5) had to be added to the necessary buffer.

#### 3.3.6. Binding Assay

After pretreatment with *R. microfusus* (see Section 3.3.1), *R. microfusus* (1 mL OD_600_ = 0.1) was mixed with 5 pmol of Cy5-labeled activated aptamer library in 500 µL of 1 × DPBS and incubated at 37 °C for 30 min. The supernatant was removed after centrifugation at 3000× *g* for 2 min, washed once, and the pellet was resuspended with 100 µL of 1 × DPBS buffer to obtain the eluted aptamers combined with cells. Finally, fluorescence intensity was measured using an Infinite M200 spectrophotometer (TECAN Trading AG, Männedorf, Switzerland) at 635 nm excitation and 670 nm emission.

### 3.4. Determination of High Specificity Aptamer Libraries

#### 3.4.1. Semi-Quantitative Analysis of *R. microfusus*

After pretreatment of all bacterial solutions (see Section 3.3.1), *R. microfusus* and control strains (including five other enteric bacteria, *B. producta*, *A. muciniphila*, *R. intestinalis*, *A. stercoricanis,* and *P. distasonis*) were mixed at different ratios. *R. microfusus* was gradually reduced from 100% to 0%. The 5 pmol activated Cy5-labeled aptamer library was then co-incubated with the bacteria in 500 µL of 1 × DPBS system for 30 min at 37 °C. After post-treatment, a comparison of the change in fluorescence intensity across the groups concerning *R. microfusus* content was made (see Section 3.3).

#### 3.4.2. Affinity Analysis

*R. microfusus* was pretreated (see Section 3.3.1). Aptamer library affinity ligation assays were performed by co-incubating different concentrations of aptamer libraries with equal amounts of *R. microfusus* (1 mL OD_600_ = 0.1) in 500 µL of 1 × DPBS at 37 °C for 30 min. Finally, the aptamer concentration was fitted to the corresponding bound fluorescence intensity by using GraphPad PRISM 8 (GraphPad Software, San Diego, CA, USA) to determine the dissociation constant (K_d_) of the fluorescent aptamer by calculating the equation Y = B_max_ × X/(K_d_ + X), where Y = measured fluorescence, Bmax = maximum fluorescence, and X = concentration of the aptamer.

#### 3.4.3. Fluorescence Microscopy

Bacterial solutions of six bacteria, *R. microfusus*, *B. producta*, *A. muciniphila*, *R. intestinalis*, *A. stercoricanis,* and *P. distasonis,* were pretreated by the above method (see Section 3.3.1). A total of 5 pmol of activated aptamer library in 500 µL 1 × DPBS was incubated separately with each bacterium (1 mL OD_600_ = 0.1) for 30 min at 37 °C. After centrifugation at 3000× *g* for 2 min, the supernatant was removed. The pellet was washed once with 500 µL of 1 × DPBS and then resuspended with 500 µL of 1 × DPBS. A total of 10 µL of each bacterial mixture was then added to slides, and fluorescence imaging of each group was obtained using a fluorescence microscope using a Leica DMi8 code (Leica Microsystems CMS GmbH, Wetzlar, Germany) at ×40 magnitude (excitation: 590–650 nm, emission: 662–738 nm) under transmitted light from a Y5 filter.

#### 3.4.4. Real-Time PCR

Eluates from SELEX rounds 1 to 13 were collected separately, and the aptamers obtained from each screening round were quantified. Samples were stained with SYBR Green I (final concentration 0.5×) (Sigma-Aldrich, St. Louis, MO, USA) and quantified in real-time by qTOWER^3^G touch (Analytik Jena GmbH, Jena, Germany) under the following thermal cycling conditions: an initial step of 3 min at 94 °C, followed by 40 cycles of 30 sec at 94 °C, 30 sec at 56 °C, and 10 sec at 72 °C. A master mix was prepared for each reaction batch using Herculase II Fusion DNA Polymerase (Agilent Technologies, Inc., Santa Clara, CA, USA) and 100 mM unmodified primers (forward primer: 5′-TAG GGA AGA GAA GGA CAT ATG AT-3′; reverse primer: 5′-TCA AGT GGT CAT GTA CTA GTC AA-3′) (biomers.net GmbH, Ulm, Germany). This was followed by a melting curve analysis consisting of 40 melting cycles, starting from 60 °C to a temperature of 95 °C. Linear regression analysis was performed to test the relationship between ddRn/dT (change in fluorescence divided by change in temperature) and the different SELEX rounds. and the Ct value were determined. Melting temperatures of the PCR products amplified in the real-time PCR reactions with aptamers eluted from the individual successive SELEX rounds as templates were used to estimate the assimilation of single nucleotide variants upon processing of the SELEX. The evolution of higher affinity in a population of aptamer sequences is supposed to result in an overall increase in the GC content within the relevant sequence space and a respective increase in the melting temperature.

### 3.5. Analysis of R. microfusus Abundance in Human Stool Samples

#### 3.5.1. Human Stool Samples

In this investigation, two healthy volunteer stool samples were utilized. Volunteers were recruited from Ulm University and signed a written informed consent form. Permission was obtained from the local ethics committee of the University of Ulm (No. 30/20). In addition, the study was designed and conducted following the regulations for using human research participants and in strict accordance with the standards set by the Declaration of Helsinki.

#### 3.5.2. Stool Bacteria Extraction

The fecal samples were weighed and vortexed in 1 × DPBS buffer for 1 min until no fecal particles were visible. The undissolved fecal pellets, as well as food residues, were then removed by filtration. The filtrate was centrifuged at 9000× *g* for 1 min, washed three times with 1 × DPBS buffer, and finally adjusted to a bacterial concentration of OD_600_ of 1.

#### 3.5.3. Analysis Based on NGS

Fecal samples were collected using INTEST.Pro (Biomes Laboratory, Wildau, Germany), which were subsequently measured and analyzed by Biomes Laboratory (Wildau, Germany) to detect fecal bacterial abundance using 16s rDNA NGS according to Lilja et al., 2021 [53].

#### 3.5.4. Analysis Based on the Aptamer Library R.m-R13

The *R. microfusus* (see Section 3.3.1) and fecal samples (see Section 3.5.2) were pretreated. *R. microfusus* (10^8^ CFU) and fecal bacteria (10^8^ CFU) were then incubated separately with 500 µL of 1 × DPBS (containing 5 pmol of activated aptamer library) for 30 min at 37 °C (see Section 3.3.2), and the fluorescence intensity was measured after elution.

## 4. Conclusions

An enriched aptamer library has successfully been evolved by a whole-cell SELEX procedure against *Rikenella microfusus* as another example of health-relevant human gut bacteria. After 13 rounds of molecular evolution and selection, the library allowed specific labeling of *R. microfusus* cells in fluorimetric binding assays and fluorescence microscopy. The discrimination of the target bacterium, *R. microfusus*, was perfectly possible from a set of five other gut bacteria as controls, with a dissociation constant below 10 nm of the aptamer library. The aptamer-based binding assay was also successful with samples directly derived from human stool samples. The library will serve as the foundation for the isolation of individual best-performing aptamers after next-generation sequencing and bioinformatic analysis. Another aim of this work is to enable the future construction and characterization of (electronic) aptasensors for the reliable and fast quantitative monitoring of individual bacterial species in the human gut microbiome.

## Figures and Tables

**Table 1:** Comparison of application relevant parameters of the proposed aptamer-based microbiome biosensor (aptamer) and existing methods (16S rRNA gene sequencing, metagenomic shotgun sequencing (MSS), and bacterial group/species quantitative polymerase chain reaction (qPCR)) for detecting and quantifying gut bacteria.

	Aptamer	16S	MSS	qPCR
Detection Time	30–60 mins	Days to weeks	Weeks to months	Hours to days
Complexity	Simple	Complex	Complex	Complex
Clinical real-time monitoring	Yes	No	No	No
Cost	Cheap	Expensive	Expensive	Expensive

**Table 2:** The initial library consists of a random sequence in the middle and a fixed sequence of primer at each end.

Name	Sequence
Random ssDNA library	5′-TAGGGAAGAGAGAGAGGACATATGAT-N_(40)_- TTTGACTAGTACATGACCACTTGA-3′
Cy5-Primer	5′-Cy5-TAGGGAAGAAGGAGAGAGATGATA-3′
Phosphate-Primer	5′-phosphate-TCAGTGTCATGTACTAGTCAA-3′

## Data Availability

Not applicable.

## Supplementary Materials

The following are available online at https://www.mdpi.com/article/10.3390/microorganisms11092266/s1, Table S1: Conditions of all SELEX rounds of *R. microfusus*, including the amount of aptamer library, counter SELEX, target SELEX, incubation conditions, washing times, and the amount of BSA/tRNA; Table S2: Next-generation sequencing results proband 1; Table S3: Next-generation sequencing results proband 2.
