# Supplementary material for: Enriched Aptamer Libraries in Fluorescence-Based Assays for Rikenella microfusus-Specific Gut Microbiome Analyses
Source: Microorganisms. 2023 Sep 9;11(9):2266. doi: 10.3390/microorganisms11092266 (PMC10535755; doi:10.3390/microorganisms11092266)
Supplement: Supplementary file 1 [file microorganisms-11-02266-s001.zip › microorganisms-2554790-supplementary.pdf]

microorganisms

Communication

Enriched Aptamer Libraries in Fluorescence-Based Assays for *Rikenella microfusus*-Specific Gut Microbiome Analyses

Supplementary Materials

Table S1. Conditions of all SELEX rounds of *R. microfusus,* including the amount of aptamer library, counter SELEX, counter SELEX times, target SELEX, washing times, and the amount of BSA/tRNA.

SELEX rounds	Aptamer (pmol)	Counter SELEX (cells)	Counter SELEX times	Target SELEX (cells)	Wash times	BSA/tRNA (pmol)
1	500	-	-	10^8	1	600
2	10	-	-	10^8	1	900
3	10	-	-	10^8	2	1200
4	10	-	-	10^8	2	1500
5	10	-	-	10^8	3	1800
6	10	2×10^7 each bacterium	1	10^8	4	2100
7	10	2×10^7 each bacterium	1	10^8	6	2400
8	5	2×10^7 each bacterium	1	10^8	6	2700
9	5	2×10^7 each bacterium	2	10^8	6	3300
10	5	2×10^7 each bacterium	2	10^8	6	3600
11	1	2×10^7 each bacterium	2	10^8	6	3900
12	1	2×10^7 each bacterium	3	10^8	6	4200
13	1	2×10^7 each bacterium	3	10^8	6	4500

1. The BSA (100mg/mL) and tRNA (10mg/mL) were incubated with *R. microfusus* to avoid non-specific binding to the cell surface.

2. Counter SELEX: Aptamer library was incubated with bacteria mix including *A. muciniphila* mucT, *A. stercoricanis*, *R. intestinalis*, *P. distasonis* and *B. producta* were co-incubated at 37℃ for 30 min

3. Target SELEX: Aptamer library was incubated with *R. microfusus* in 37℃ for 30 min.

Table S2. *Rikenella* abundance in fecal samples of proband 1 determined by 16S rRNA NGS.

Kingdom	Phylum	Class	Order	Family	Genus	Species	Absolute _counts	Relative _counts
Bacteria	Bac-teroidetes	Bacte-roidia	Bacteroi-dales	Rikenel-laceae	Alistipes	finegoldii	2.76	6.01096×10^{-3}
Bacteria	Bac-teroidetes	Bacte-roidia	Bacteroi-dales	Rikenel-laceae	Alistipes	onderdonkii WAL 8169 = DSM 19147	10.83	2.35865×10^{-2}
Bacteria	Bac-teroidetes	Bacte-roidia	Bacteroi-dales	Rikenel-laceae	Alistipes	putredinis DSM 17216	42.85	9.33224×10^{-2}
Bacteria	Bac-teroidetes	Bacte-roidia	Bacteroi-dales	Rikenel-laceae	Alistipes	Alisti-pes;shahii WAL 8301	10.11	2.20184×10^{-2}
Bacteria	Bac-teroidetes	Bacte-roidia	Bacteroi-dales	Rikenel-laceae	Alistipes	Unspecific _Alistipes	89.45	1.94812×10^{-1}
Bacteria	Bac-teroidetes	Bacte-roidia	Bacteroi-dales	Rikenel-laceae	Unspecific _Rikenel-laceae	Unspecific _Rikenel-laceae	2.0	4.35577×10^{-3}

Table S3. *Rikenella* abundance in fecal samples of proband 2 determined by 16S rRNA NGS.

Kingdom	Phylum	Class	Order	Family	Genus	Species	Absolute _counts	Relative _counts
Bacteria	Bacteroidetes	Bacteroidia	Bacteroidales	Rikenellaceae	Alistipes	finegoldii	0.78	9.16639×10^{-4}
Bacteria	Bacteroidetes	Bacteroidia	Bacteroidales	Rikenellaceae	Alistipes	inops	77.83	9.14641×10^{-2}
Bacteria	Bacteroidetes	Bacteroidia	Bacteroidales	Rikenellaceae	Alistipes	onderdonkii WAL 8169 = DSM 19147	1.0	1.17518×10^{-3}
Bacteria	Bacteroidetes	Bacteroidia	Bacteroidales	Rikenellaceae	Alistipes	putredinis DSM 17216	1.77	2.08006×10^{-3}
Bacteria	Bacteroidetes	Bacteroidia	Bacteroidales	Rikenellaceae	Alistipes	shahii WAL 8301	297.49	3.49604×10^{-1}
Bacteria	Bacteroidetes	Bacteroidia	Bacteroidales	Rikenellaceae	Alistipes	Unspecific _Alistipes	554.0	$6.51048E \times 10^{-1}$
Bacteria	Bacteroidetes	Bacteroidia	Bacteroidales	Rikenellaceae	DMER64	Unspecific _DMER64	1.0	1.17518×10^{-3}
Bacteria	Bacteroidetes	Bacteroidia	Bacteroidales	Rikenellaceae	Unspecific _Rikenellaceae	Unspecific _Rikenellaceae	0.5	5.87589×10^{-4}
